# Evidence-based perioperative diagnosis and management of pulmonary embolism: A systematic review

**DOI:** 10.1016/j.amsu.2022.103684

**Published:** 2022-04-28

**Authors:** Lamesgen Geta Abate, Samuel Debas Bayable, Melaku Bantie Fetene

**Affiliations:** a Department of Anaesthesia, College of Medicine and Health Science, Debre Markos University, Debre Markos, Ethiopia; b Department of Anaesthesia, College of Medicine and Health Science, Debre Berhan University, Debre Berhan, Ethiopia; c Advanced Clinical Anesthesia and Critical Care, Ethiopia

**Keywords:** Pulmonary embolism, Anesthesia management, Anticoagulation, Thrombolysis, CTPA, Computed Tomography Pulmonary Angiography, CASP, Critical Appraisal Skills Programmed, DVT, Deep Venous Thrombosis, PE, Pulmonary Embolism, WHO, World Health Organization

## Abstract

**Background:**

The diagnosis and treatment of pulmonary embolism have multi-modal approach based on specificity, sensitivity, availability of the machine, and associated risks of imaging modalities.

**Aim:**

This review aimed to provide shreds of evidence that improve perioperative diagnosis and management of suspected pulmonary embolism.

**Methods:**

The study was conducted in accordance with the Preferred Reporting Items for Systematic Reviews and Meta-Analyses (PRISMA) guideline 2020. After a clear criteria has been established an electronic searching database was conducted using PubMed, Google Scholar, Cochrane library, and Cumulative Index of Nursing and Allied Health Literature (CINAHL), with Key search terms included:(‘pulmonary embolism’ AND′ anesthesia management ‘, ‘anticoagulation’ AND ‘pulmonary embolism’, ‘thrombolysis ‘AND ‘pulmonary embolism’, ‘surgery’ AND′ pulmonary embolism’), were used to draw the evidence.

The quality of literatures were categorized based on WHO 2011 level of evidence and degree of recommendation, in addition, the study is registered with research registry unique identifying number (UIN) of reviewregistry1318.” and has high quality based on AMSTAR2 assessment criteria.

**Results:**

A totally of 27 articles were included [guidelines (n = 3), Cochrane (=5), systemic reviews (n = 7), meta-analyses (=2), RCT (n = 4), cohort studies (n = 3), and cross-sectional study (n = 3) and illegible articles identified from searches of the electronic databases were imported into the ENDNOTE software version X7.1 and duplicates were removed.

**Discussion:**

Currently divergent and contradictory approaches are implemented in diagnosis and management for patients suspected of pulmonary embolism.

**Conclusion:**

All perioperative patients, especially trauma victims, prostate or orthopedic surgery, malignancy, immobility, and obesity; smokers; and oral contraceptive users, antipsychotic medications are at increased risk of venous thromboembolism and need special caution during surgery and anesthesia**.**

## Highlights

• Currently divergent and contradictory approaches are implemented in diagnosis and management for patients suspected of pulmonary embolism.
• This study improves perioperative diagnosis and management of suspected pulmonary embolism patients.
• Patients having trauma, orthopedic surgery, malignancy, immobility; and being smoker; are at risk of VTE and need special caution during surgery.

## 1 Introduction

Pulmonary Embolism(PE) is a treatable illness caused by the migration of thrombi to the pulmonary circulation, from the veins of the lower extremities[[1], [2], [3], [4]], commonly arises from Deep veins of the legs which range from asymptomatic, to massive which results in sudden death[5].

The prevalence of pulmonary embolism in developed countries was about 2.2% [6,7].and in the United States it causes a high rank among cardiovascular mortality[7], while in Africa, it has been reported in 3.8–32.4%, in patients with clinical suspicion of pulmonary embolism(PE) [4], but the incidence of PE increased to fivefold during and after surgery[8]. even though the diagnosis of PE is often obscured intraoperatively with common disorders including bleeding and infection physicians and anesthetists are responsible for the diagnosis and management of such fatal disorders [8].

Pulmonary embolism-associated vasoconstriction, mediated by the release of thromboxane A2 and serotonin, contributes to the initial increase in pulmonary vascular resistance (PVR) after PE. Anatomical obstruction and hypoxic vasoconstriction in the affected lung area lead to an increase in PVR and a proportional decrease in arterial compliance[7, 9]. Helical computed tomography and Transesophageal echocardiography are preferred to diagnose in the operating room for all patients at increased risk of venous thromboembolism, such as trauma victims and those undergoing prostate or orthopedic surgery [6,8].

The initial management of pulmonary embolism may be started before a definitive diagnosis is established, started with supportive treatment followed by vasopressors aimed at stabilizing the patient and minimizing the effect of the embolic occlusion to improve right ventricular function and contract the systemic vasculature to maintain blood pressure respectively[8, 10].

## 2 Justification

Pulmonary embolism is a potentially life-threatening condition that needs immediate diagnosis and management [9], since Surgery puts patients at a fivefold increased risk for pulmonary embolism[[7], [8], [9]], in addition, perioperative thromboprophylaxis is underutilization in Ethiopian hospital ward patients who have a risk of pulmonary embolism and professionals do not adhere to guideline recommendations[11].

Even if Pulmonary angiography is the standard for establishing the presence of pulmonary embolism, a negative pulmonary angiogram doesn't rule out pulmonary embolism due to its insufficient sensitivity to detect small emboli [8,9,12]. In addition, D-dimer tests are rapid, simple, inexpensive, and can prevent high costs associated with expensive diagnostic tests [13]. Although pulmonary embolism is a leading cause of death worldwide, controversies' regarding diagnosis, treatment, and follow-up persist, having a wide range of treatment options including anticoagulation alone, catheter-directed thrombolysis, catheter embolectomy, surgical embolectomy, and/or mechanical circulatory support device, so this study helps to develop an institutional working protocol to provide optimal diagnosis and treatment of pulmonary embolism during the perioperative period of high risk and suspected patients.

## 3 Objectives

### 3.1 General objective

To improve perioperative diagnosis and management of suspected pulmonary embolism patients.

### 3.2 Specific objectives

To provide a working framework for diagnosis of pulmonary embolism.

To prepare pulmonary embolism management protocol.

## 4 Methods

**Fig. 1:**
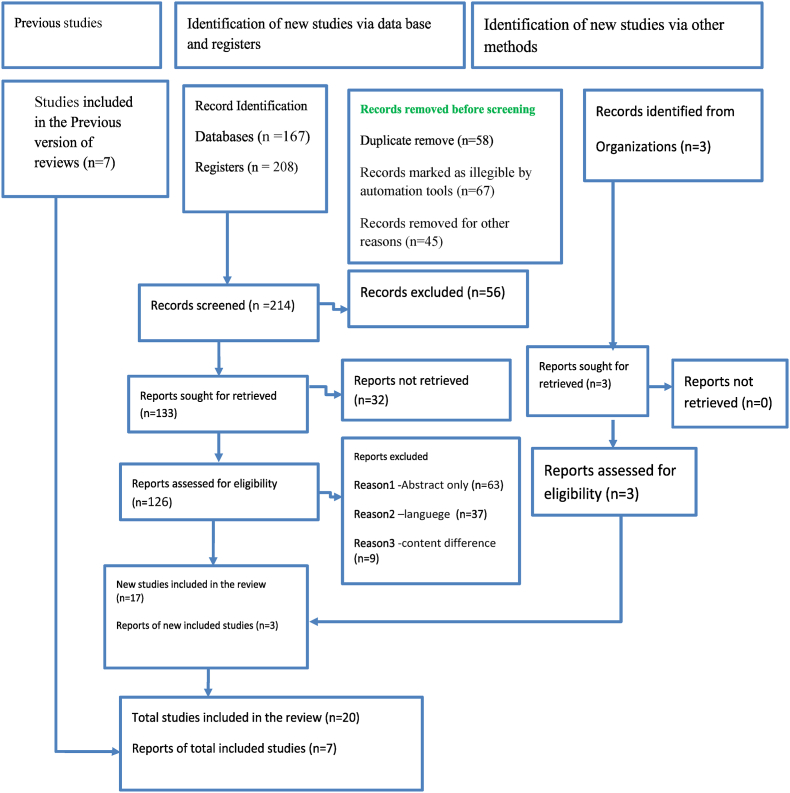
Flow chart for selection of studies using 2020 PRISMA flow diagram.

The study is conducted in accordance with the Preferred Reporting Items for Systematic Reviews and Meta-Analyses (PRISMA) guideline 2020 [14] as shown in (Fig. 1). After a clear criteria has been established an electronic database search was conducted using PubMed, Google Scholar, Cochrane Library, Cumulative Index of Nursing and Allied Health Literature (CINAHL), with Key search terms included:(‘pulmonary embolism’ AND′ anesthesia management ‘, ‘anticoagulation’ AND ‘pulmonary embolism’, ‘thrombolysis ‘AND ‘pulmonary embolism’, ‘surgery’ AND’ pulmonary embolism’), were used to draw pieces of evidence. Synonyms and truncations of these keywords were used, and database-specific medical subject headings (MeSH) were also included. The inclusion and exclusion of these studies are stated in (Table 1).

**Fig. 2:**
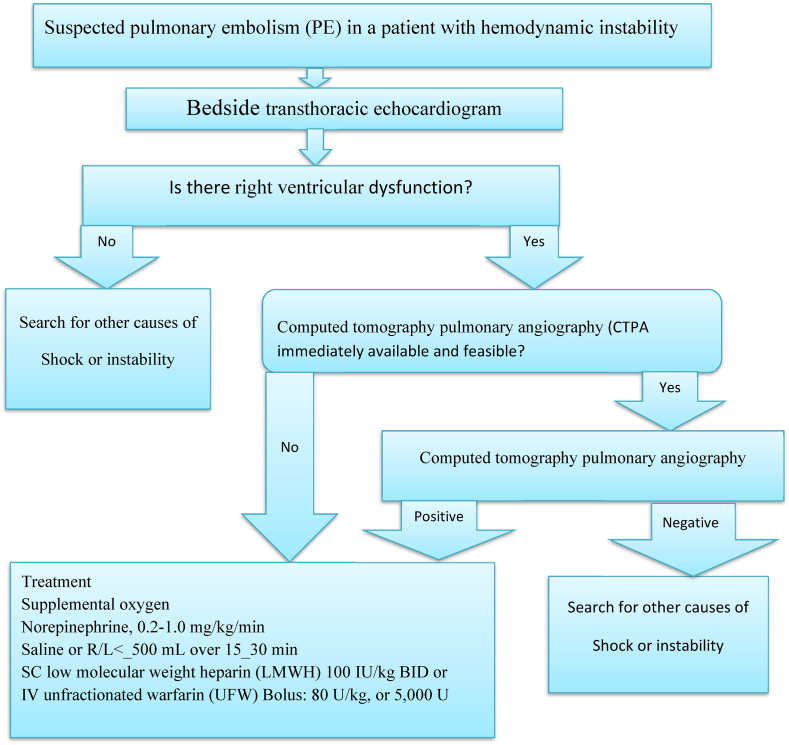
Algorithm for patients with suspected high-risk pulmonary embolism presenting with hemodynamic instability.

**Table 1:** The inclusion and exclusion criteria of the studies in this review.

	Inclusion criteria	Exclusion criteria
Population	Surgical patients at risk of pulmonary embolism	Surgical patient having less risk factor for pulmonary embolism
Sources	Published in peer reviewed Journals	Not published in peer reviewed Journals
Publication date	Published between 2010 and 2022	Published prior to 2010
Language	Published in English	Published in languages other than English
Availability	Full text	Full text unavailable
Study design	Primary research that focuses on pulmonary embolism	Secondary research
Quality	Studies evaluated as moderate or high quality according to the Critical Appraisal Skills Programme checklists	Studies evaluated as low quality according to the Critical Appraisal Skills Programme checklists

Selection of studies was detailed using PRISMA 2020 flow diagram(14) as shown (Fig. 1).

### 4.1 Data quality appraisal and synthesis

Before inclusion each study in the review of the literature, the quality of each study was assessed by all authors independently using the Critical Appraisal Skills Programme (CASP) checklists [15,16],and authors consulted their findings with each other, then agreed on the final studies to be included in the review. The authors defined moderate and high methodological quality as meeting 60–80% and 90–100% of the CASP checklist criteria respectively [[15], [16], [17]]. The minimum percentage threshold for inclusion in the review of the literature was decided to be 60% of the criteria [17] and uses the WHO 2011 level of evidence and degree of recommendation (Table 2) [18], and this study is registered with a link of https://www.researchregistry.com/browse-the-registry#registryofsystematicreviewsmeta-analyses/with a unique identifying number (UIN) of 1318 and the study has high quality based on AMSTAR 2 quality assessment checklist/https://amstar.ca/Amstar_Checklist.php.

**Table 2:** WHO 2011 level of evidence and degree of recommendation.

Level	Type of evidence	Degree of recommendation
1a	Meta-analyses, systematic reviews of RCTs	Strongly recommended/directly applicable
1b	Systematic review	Highly recommended/directly applicable
1c	Randomized clinical trials/RCTs	Recommended/applicable
2a	Systematic reviews of case-control or cohort studies.	Extrapolated evidence from other studies
3a	Non-analytic studies, e.g. case reports, case series	Extrapolated evidence from other studies

## 5 Results

A summary of the included studies in the review of the literature can be seen in (Table 1). Totally 27 articles were included [guidelines (n = 3), Cochrane (=5), systemic reviews (n = 7), meta-analyses (=2), RCT (n = 4), cohort studies (n = 3), and cross-sectional study (n = 3) which were more updated and focused on pulmonary embolism management. Illegible articles identified from searches of the electronic databases were imported into the ENDNOTE software version X7.1 (Tomson Reuters, USA) and duplicates were removed. Before findings had begun, full-length articles of the selected studies were read to confirm for fulfilling the inclusion criteria.

## 6 Discussion

Pulmonary embolism (PE) is a life-threatening condition in which a clot travels from deep veins of the lower extremity to the circulation and lodges into the lungs[7]. Clinical presentation of venous thromboembolism (VTE) is globally the third most frequent acute cardiovascular syndrome behind myocardial infarction and stroke [7].

Human immunodeficiency virus (HIV) increases the risk of PE two-to ten fold as compared with the general population, major surgery, Hip or knee replacement, and General anesthesia when compared with epidural [4,19].

Computed Tomography Pulmonary Angiography (CTPA) has greatly improved the diagnostic approach to patients with suspected PE and is considered to be the reference imaging test, but should be used, with caution in some patients, such as patients with severe renal insufficiency, those with known allergy to contrast media, and pregnant women[[19], [20], [21]]. Additionally, ECG findings include sinus tachycardia, atrial dysrhythmia, dramatic shift in R wave axis, incomplete or complete right bundle-branch block, inferolateral ST-segment elevation, or depression, inversion of T waves in leads V1–V4, and biomarkers such as elevated D-dimers or fibrin degradation are suggestive of PE [9,13,19,22,23]. D-dimer assays can rule out PE. But has low specificity of positive tests, especially in older age groups[13, 24].

If the patient presents with at least three parameters out of the five most common signs and symptoms of PE (cough, hypoxia, dyspnea, tachycardia, and chest pain) with the inclusion of X-ray and echocardiography results it is satisfactory pieces of evidence to make high suspicions of acute pulmonary embolism that requires diagnosis and management at bedside within a few minutes, but if the patient is hemodynamically stable, CTPA can be performed to confirm the diagnosis(12, 25).

Acute pulmonary embolism requires anticoagulation to prevent early death and recurrent symptomatic fatal venous thromboembolism. The standard duration of anticoagulation cover at least 3 months and parenteral anticoagulation [unfractionated heparin, low molecular weight heparin, or fondaparinux] over the first 5–10 days should be given to treat acute PE [7,19,24,26,27].

Hypoxaemia is one feature of severe PE and resulted from the mismatch between ventilation and perfusion, so supplemental oxygen is required in patients' level of SpaO2 <90%. Patients with right ventricular failure are highly susceptible to the development of severe hypotension during induction of anesthesia, intubation, and positive-pressure ventilation [7]. Thrombolytic therapy is associated with a significant reduction in overall mortality, pulmonary embolism recurrence as compared with heparin, but increased intracranial hemorrhage and is not significant in hemodynamically stable patients [8,10,19,28,29].

Hemodynamically deteriorating suspected pulmonary embolism patients require rescue thrombolytic therapy, in addition, surgical embolectomy and catheter-directed treatment are alternatives to the treatment of pulmonary embolism to rescue thrombolytic therapy [7,9,19,24]. Intravenous catheter filters reduce the risk of subsequent pulmonary embolism, increase the risk of DVT, and have no significant effect on overall mortality, so Intravenous catheter (IVC) filters should be considered for limited scenarios, such as contraindication to antithrombotic therapy or recurrent pulmonary embolism despite adequate anticoagulation[7, 30]. A fixed-dose regimen of rivaroxaban is as effective as standard anticoagulant therapy for the treatment of DVT prophylaxis, without the need for laboratory monitoring[28, 31].

**Fig. 3:**
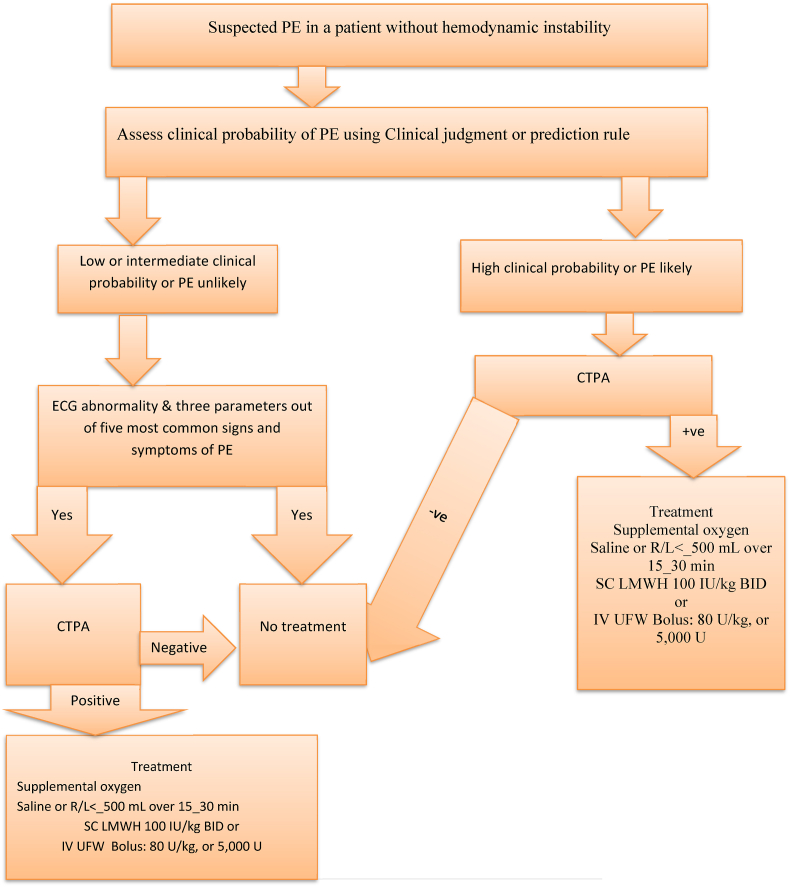
Algorithm for patients with suspected pulmonary embolism without hemodynamic instability.

Deep venous thrombosis (DVT) can be prevented through non-pharmacologic prophylaxis (compression stockings, leg elevation, sequential compression devices (SCDs), ambulation, and vena cava filter) and pharmacologic intervention, which is through the use of blood-thinning medications[2, 11, 27]. The most common blood thinner prophylaxis in Ethiopia is unfractionated heparin (UFH) and warfarin. The major side effect of blood-thinning medications is an increased risk of bleeding and some patients are contraindicated for blood-thinning medications since they have a greater risk of developing adverse events[11, 24]. The overall mortality rate in untreated patients is 30%, with approximately 10% of patients dying within 1 h of the event. Haemodynamically unstable patients have the highest mortality rate, which can be as high as 58% [4,7,32]. Generally the overall summary for patients with suspected high-risk pulmonary embolism presenting with hemodynamic **stable** and **instable** patients are detailed as shown in (Fig. 2 and Fig. 3) respectively.

## 7 Conclusion

All perioperative patients, especially trauma victims, prostate or orthopedic surgery, malignancy, immobility, and obesity; smokers; and oral contraceptive users, antipsychotic medications are at increased risk of venous thromboembolism and need special caution during surgery and anesthesia**.**

## Authors contribution

•Lamesgen Geta Abate = study concept or design, litreture review, data cleaning, and data interpretation.

•Samuel Debas Bayable = litreture review, data cleaning, and data interpretation, manuscript wrting

•Melaku Bantie Fetene = Check grammer and plagiarism,corrected flow of ideas.

## Registration of research studies

1. Name of the registry: **Research studies**.

2. Unique Identifying number or registration ID: 1318.

3. Hyperlink to your specific registration (must be publicly accessible and will be checked): researchregistry1318”.

## Guarantor

Samuel Debas Bayable, and Lamesgen Geta Abate.

## Ethics approval and consent to participate

Ethical approval was granted with Debre Markos University School of Medicine.

## Availability of data and materials

Data and materials are fully available without restriction and present upon request.

## Funding

The authors received no financial support for the research authorship/or publication of this article**.**

## Consent

Not required.

## Provenance and peer review

All authors were equally participated to address Comments and suplimented in the manuscript before sending the revised form for publication.

Not commissioned, externally peer, reviewer.

Kokeb Desta (Asst.prof. in Advanced Clinical Anesthesia) kokeb.desta@gmail.com helps us in method of reviewing and overall writings in spelling, grammar and punctuation.

## Declaration of competing interest

The authors declare that there is no conflict of interest.

## Acronyms and abbreviation

CTPA: Computed Tomography Pulmonary Angiography
CASP: Critical Appraisal Skills Programmed
DOACs: Direct Oral Anticoagulants
DVT: Deep Venous Thrombosis
PE: Pulmonary Embolism
WHO: World Health Organization

## References

[1] Bajc M., Schümichen C., Grüning T., Lindqvist A., Le Roux P.-Y., Alatri A. (2019). EANM guideline for ventilation/perfusion single-photon emission computed tomography (SPECT) for diagnosis of pulmonary embolism and beyond. Eur. J. Nucl. Med. Mol. Imag..

[2] Doundoulakis I., Antza C., Karvounis H., Giannakoulas G. (2020). Non-vitamin K antagonist oral anticoagulants in pulmonary embolism: an overview of systematic reviews. Curr. Pharmaceut. Des..

[3] Robertson L., Kesteven P., McCaslin J.E. (2015). Oral direct thrombin inhibitors or oral factor Xa inhibitors for the treatment of pulmonary embolism. Cochrane Database Syst. Rev..

[4] Ramlakhan R., Rajkumar A., Andronikou S. (2017). The prevalence and radiological findings of pulmonary embolism in HIV-positive patients referred for computed tomography pulmonary angiography in the Western Cape of South Africa. Cardiovasc. J. Africa.

[5] Herrería Palacios P. (2020).

[6] Raynal P.-A., Cachanado M., Truchot J., Damas-Perrichet C., Feral-Pierssens A.-L., Goulet H. (2019). Prevalence of pulmonary embolism in emergency department patients with isolated syncope: a prospective cohort study. Eur. J. Emerg. Med..

[7] Konstantinides S.V., Meyer G., Becattini C., Bueno H., Geersing G.-J., Harjola V.-P. (2020). 2019 ESC Guidelines for the diagnosis and management of acute pulmonary embolism developed in collaboration with the European Respiratory Society (ERS) the Task Force for the diagnosis and management of acute pulmonary embolism of the European Society of Cardiology (ESC). Eur. Heart J..

[8] Desciak M.C., Martin D.E. (2011). Perioperative pulmonary embolism: diagnosis and anesthetic management. J. Clin. Anesth..

[9] Lavorini F., Di Bello V., De Rimini M.L., Lucignani G., Marconi L., Palareti G. (2013). Diagnosis and treatment of pulmonary embolism: a multidisciplinary approach. Multidiscipl. respirat. med..

[10] Hao Q., Dong B., Yue J., Wu T., Liu G. (2015). Thrombolytic therapy for pulmonary embolism. Cochrane Database Syst. Rev..

[11] Ayalew M.B., Horsa B.A., Zeleke M.T. (2018). Appropriateness of pharmacologic prophylaxis against deep vein thrombosis in medical wards of an Ethiopian referral hospital. Int. J. Vasc. Med..

[12] Schellhaaß A., Walther A., Konstantinides S., Böttiger B.W. (2010). The diagnosis and treatment of acute pulmonary embolism. Deutsches Ärzteblatt Int..

[13] Crawford F., Andras A., Welch K., Sheares K., Keeling D., Chappell F.M. (2016). D‐dimer test for excluding the diagnosis of pulmonary embolism. Cochrane Database Syst. Rev..

[14] Page M.J., McKenzie J.E., Bossuyt P.M., Boutron I., Hoffmann T.C., Mulrow C.D. (2021). The PRISMA 2020 statement: an updated guideline for reporting systematic reviews. Int. J. Surg..

[15] CASP U. (2013).

[16] Casp U. (2017). Critical appraisal skills programme (CASP). Qualit. res. checklist.

[17] Horntvedt M.-E.T., Nordsteien A., Fermann T., Severinsson E. (2018). Strategies for teaching evidence-based practice in nursing education: a thematic literature review. BMC Med. Educ..

[18] Brożek J., Akl E., Compalati E., Kreis J., Terracciano L., Fiocchi A. (2011). Grading quality of evidence and strength of recommendations in clinical practice guidelines part 3 of 3. The GRADE approach to developing recommendations. Allergy.

[19] Lapner S.T., Kearon C. (2013). Diagnosis and management of pulmonary embolism. Bmj.

[20] Antic D., Lefkou E., Otasevic V., Banfic L., Dimakakos E., Olinic D. (2022). Position paper on the management of pregnancy-associated superficial venous thrombosis. Balkan working group for prevention and treatment of venous thromboembolism. Clinic. Appl. Thromb./Hemostasis..

[21] Hirsh J., Hoak J. (1996). Management of deep vein thrombosis and pulmonary embolism: a statement for healthcare professionals from the council on thrombosis (in consultation with the council on cardiovascular radiology), American Heart Association. Circulation.

[22] Somasundaram K., Ball J. (2013). Medical emergencies: pulmonary embolism and acute severe asthma. Anaesthesia.

[23] Mclintock C., Brighton T., Chunilal S., Dekker G., Mcdonnell N., Mcrae S. (2012). Recommendations for the diagnosis and treatment of deep venous thrombosis and pulmonary embolism in pregnancy and the postpartum period. Aust. N. Z. J. Obstet. Gynaecol..

[24] Tapson V.F. (2012). Advances in the diagnosis and treatment of acute pulmonary embolism. F1000 med. rep..

[25] Shonyela F.S., Yang S., Liu B., Jiao J. (2015). Postoperative acute pulmonary embolism following pulmonary resections. Ann. Thorac. Cardiovasc. Surg..

[26] Members A.T.F., Konstantinides S.V., Torbicki A., Agnelli G., Danchin N., Fitzmaurice D. (2014). 2014 ESC guidelines on the diagnosis and management of acute pulmonary embolism: the task force for the diagnosis and management of acute pulmonary embolism of the European society of cardiology (ESC) endorsed by the European respiratory society (ERS). Eur. Heart J..

[27] Cutts B.A., Dasgupta D., Hunt B.J. (2013). New directions in the diagnosis and treatment of pulmonary embolism in pregnancy. Am. J. Obstet. Gynecol..

[28] Marti C., John G., Konstantinides S., Combescure C., Sanchez O., Lankeit M. (2015). Systemic thrombolytic therapy for acute pulmonary embolism: a systematic review and meta-analysis. Eur. Heart J..

[29] Birhan A., Assefa T., Beyene A., Ndayishimiye P., Woldu M.A. (2019). Outcome of acute deep venous thrombosis using standard treatment versus thrombolytics: a literature review. Int. J. Hematol. Oncol. Stem Cell Res..

[30] Bikdeli B., Chatterjee S., Desai N.R., Kirtane A.J., Desai M.M., Bracken M.B. (2017). Inferior vena cava filters to prevent pulmonary embolism: systematic review and meta-analysis. J. Am. Coll. Cardiol..

[31] Investigators E.P. (2012). Oral rivaroxaban for the treatment of symptomatic pulmonary embolism. N. Engl. J. Med..

[32] Liew J., Stevens J., Slatore C. (2018). Refractory hypoxemia in a patient with submassive pulmonary embolism and an intracardiac shunt: a case report and review of the literature. Perm. J..

