# Supplementary figures and images for: Evidence-based perioperative diagnosis and management of pulmonary embolism: A systematic review
Source: Ann Med Surg (Lond). 2022 Apr 28;77:103684. doi: 10.1016/j.amsu.2022.103684 (PMC9142630; doi:10.1016/j.amsu.2022.103684)

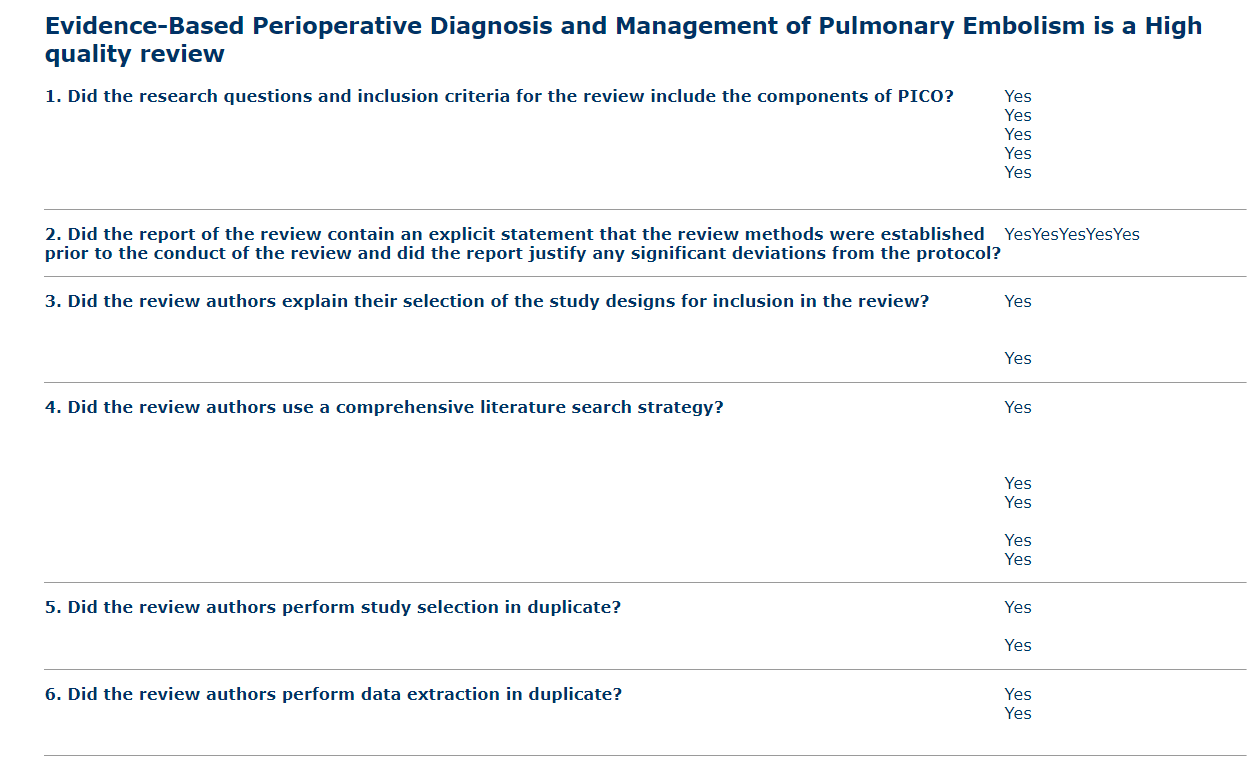


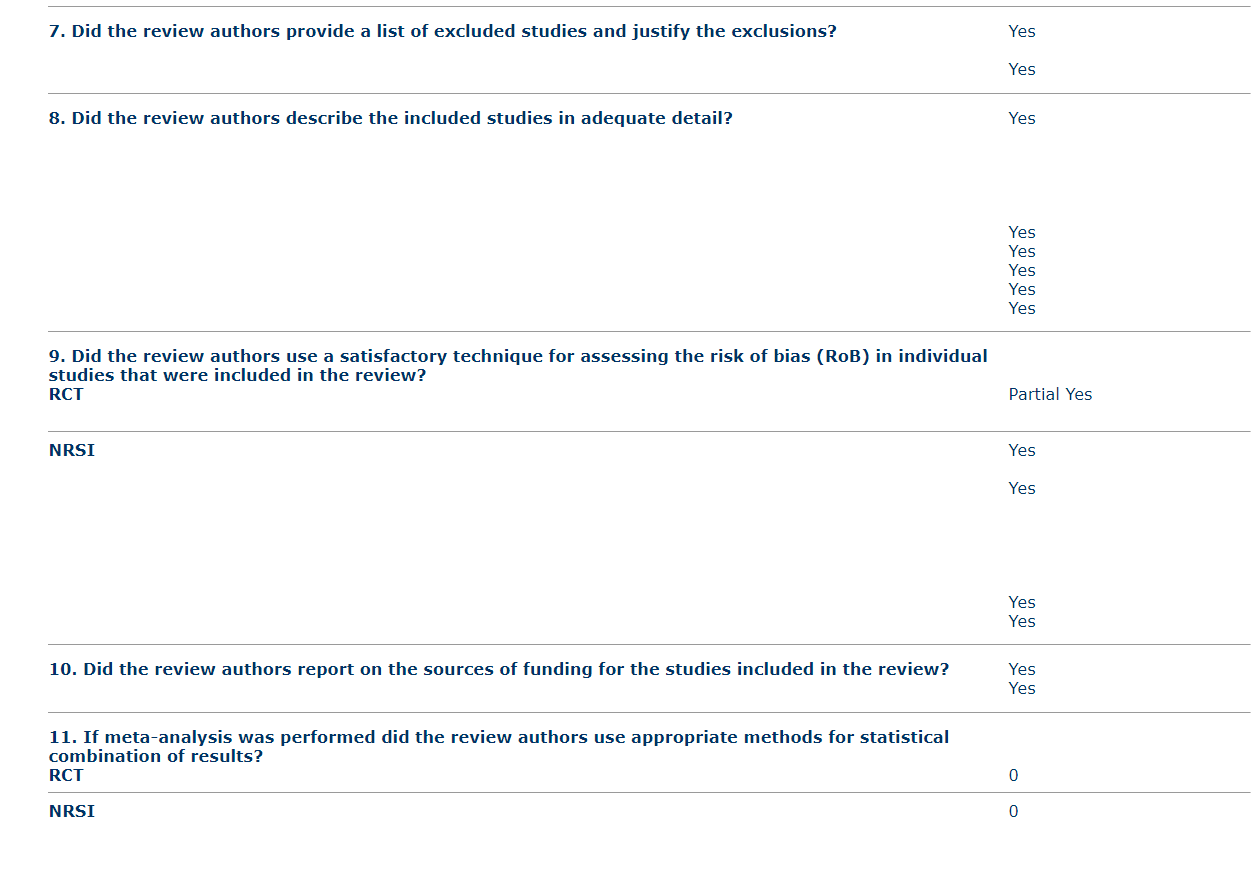


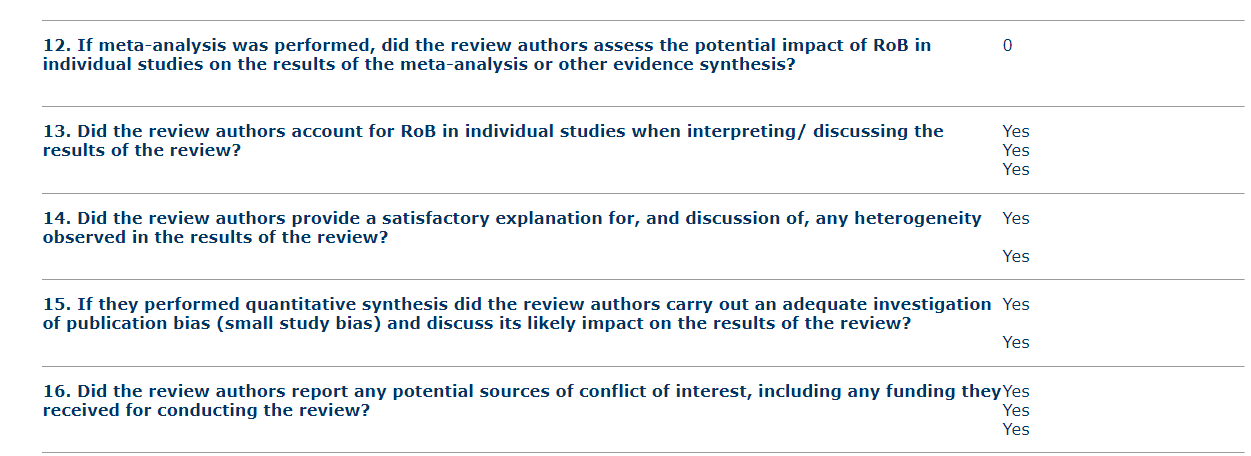

Supplement: Multimedia component 1 [file mmc1.docx]
