# Supplementary material for: Evidence-based perioperative diagnosis and management of pulmonary embolism: A systematic review
Source: Ann Med Surg (Lond). 2022 Apr 28;77:103684. doi: 10.1016/j.amsu.2022.103684 (PMC9142630; doi:10.1016/j.amsu.2022.103684)
Supplement: Multimedia component 2 [file mmc2.docx]

**Annex III: 2020 PRISMA check list**

| Section/topic | | # | | | | Checklist item | page # |
| --- | --- | --- | --- | --- | --- | --- | --- |
| TITLE | | | | | | |  |
| Title | | 1 | | | | Evidence-Based Perioperative Diagnosis and Management of Pulmonary Embolism: - A Systematic Review | 1 |
| ABSTRACT | | | | | | |  |
| Structured summary | | | 2 | | **Background:** The diagnosis and treatment of pulmonary embolism have ~~a~~ multi-modal approach based on specificity, sensitivity, availability of the machine, and associated risks of imaging modalities.  **Aim: -** This review aimed to provide shreds of evidence that improve perioperative diagnosis and management of suspected pulmonary embolism.  **Methods:** The study was conducted in accordance with the Preferred Reporting Items for Systematic Reviews and Meta-Analyses (PRISMA) guideline 2020. After a clear criteria has been established an electronic searching database was conducted using PubMed, Google Scholar, Cochrane library, and Cumulative Index of Nursing and Allied Health Literature (CINAHL), with Key search terms included:(‘pulmonary embolism’ AND’ anesthesia management ’, ‘anticoagulation’ AND ‘pulmonary embolism’, ‘thrombolysis ‘AND ‘pulmonary embolism’, ‘surgery’ AND’ pulmonary embolism’), were used to draw the evidence.  The quality of literatures were categorized based on WHO 2011 level of evidence and degree of recommendation, in addition, the study is registered with research registry unique identifying number (UIN) of [reviewregistry1318](Manuscript%20on%20%20PE.docx).” and has high quality based on AMSTAR2 assessment criteria.  **Results**: -. A totally of 27 articles were included [guidelines (n=3), Cochrane (=5), systemic reviews (n=7), meta-analyses (=2), RCT (n=4), cohort studies (n=3), and cross-sectional study (n=3) and illegible articles identified from searches of the electronic databases were imported into the ENDNOTE software version X7.1 and duplicates were removed.  **Discussion:** Currently divergent and contradictory approaches are implemented in diagnosis and management for patients suspected of pulmonary embolism.  **Conclusion:** All perioperative patients, especially trauma victims, prostate or orthopedic surgery, malignancy, immobility, and obesity; smokers; and oral contraceptive users, antipsychotic medications are at increased risk of venous thromboembolism and need special caution during surgery and anesthesia | | 2 |
| **Key words:** | | | 2 | | pulmonary embolism, anesthesia management, anticoagulation, and thrombolysis | | 2 |
| INTRODUCTION | | | | | | |  |
| Background | | | | 3  3 | Pulmonary Embolism(PE) is a treatable illness caused by the migration of thrombi to the pulmonary circulation, from the veins of the lower extremities(1-4), commonly arises from Deep veins of the legs which range from asymptomatic, to massive which results in sudden death(5).  The prevalence of pulmonary embolism in developed countries was about 2.2%(6, 7).and in the United States it causes a high rank among cardiovascular mortality(7), while in Africa, it has been reported in 3.8-32.4%, in patients with clinical suspicion of pulmonary embolism(PE) (4), but the incidence of PE increased to fivefold during and after surgery(8). even though the diagnosis of PE is often obscured intraoperatively with common disorders including bleeding and infection physicians and anesthetists are responsible for the diagnosis and management of such fatal disorders (8).  Pulmonary embolism-associated vasoconstriction, mediated by the release of thromboxane A2 and serotonin, contributes to the initial increase in pulmonary vascular resistance (PVR) after PE. Anatomical obstruction and hypoxic vasoconstriction in the affected lung area lead to an increase in PVR and a proportional decrease in arterial compliance(7, 9). Helical computed tomography and Transesophageal echocardiography are preferred to diagnose in the operating room for all patients at increased risk of venous thromboembolism, such as trauma victims and those undergoing prostate or orthopedic surgery (6, 8).  The initial management of pulmonary embolism may be started before a definitive diagnosis is established, started with supportive treatment followed by vasopressors aimed at stabilizing the patient and minimizing the effect of the embolic occlusion to improve right ventricular function and contract the systemic vasculature to maintain blood pressure respectively(8, 10). | | 3 |
| Justification | | | | 3&4 | Pulmonary embolism is a potentially life-threatening condition that needs immediate diagnosis and management (9), since Surgery puts patients at a fivefold increased risk for pulmonary embolism(7-9), in addition, perioperative thromboprophylaxis is underutilization in Ethiopian hospital ward patients who have a risk of pulmonary embolism and professionals do not adhere to guideline recommendations(11).  Even if Pulmonary angiography is the standard for establishing the presence of pulmonary embolism, a negative pulmonary angiogram doesn’t rule out pulmonary embolism due to its insufficient sensitivity to detect small emboli (8, 9, 12). In addition, D-dimer tests are rapid, simple, inexpensive, and can prevent high costs associated with expensive diagnostic tests (13). Although pulmonary embolism is a leading cause of death worldwide, controversies’ regarding diagnosis, treatment, and follow-up persist, having a wide range of treatment options including anticoagulation alone, catheter-directed thrombolysis, catheter embolectomy, surgical embolectomy, and/or mechanical circulatory support device, so this study helps to develop an institutional working protocol to provide optimal diagnosis and treatment of pulmonary embolism during the perioperative period of high risk and suspected patients. | | 2 |
| Objectives | | | | | | |  |
| This review of the literature aimed to provide a synthesis of the evidence pertaining to improve perioperative diagnosis and management of suspected pulmonary embolism patients. | | | | | | | 1 |
| Methods | 4 | | | The study is conducted in accordance with the Preferred Reporting Items for Systematic Reviews and Meta-Analyses (PRISMA) guideline 2020 (14) as shown in (**Figure1**). After a clear criteria has been established an electronic database search was conducted using PubMed, Google Scholar, Cochrane Library, Cumulative Index of Nursing and Allied Health Literature (CINAHL), with Key search terms included:(‘pulmonary embolism’ AND’ anesthesia management ’, ‘anticoagulation’ AND ‘pulmonary embolism’, ‘thrombolysis ‘AND ‘pulmonary embolism’, ‘surgery’ AND‘ pulmonary embolism’), were used to draw pieces of evidence. Synonyms and truncations of these keywords were used, and database-specific medical subject headings (MeSH) were also included. The inclusion and exclusion of these studies are stated in (**Table 1**).  **Table 1.** The inclusion and exclusion criteria of the studies in this review.   \|  \| Inclusion criteria \| Exclusion criteria \| \| --- \| --- \| --- \| \| Population \| Surgical patients at risk of pulmonary embolism \| Surgical patient having less risk factor for pulmonary embolism \| \| Sources \| Published in peer reviewed  Journals \| Not published in peer reviewed  Journals \| \| Publication date \| Published between 2010 and 2022 \| Published prior to 2010 \| \| Language \| Published in English \| Published in languages other than  English \| \| Availability \| Full text \| Full text unavailable \| \| Study design \| Primary research that focuses on pulmonary embolism \| Secondary research \| \| Quality \| Studies evaluated as moderate or  high quality according to the  Critical Appraisal Skills Programme checklists \| Studies evaluated as low quality  according to the Critical Appraisal  Skills Programme checklists \|   Selection of studies was detailed using PRISMA 2020 flow diagram(14) as shown (**Figure 1**).  Identification of new studies via data base and registers  Identification of new studies via other methods    Previous studies  Record Identification  Databases (n =167)  Registers (n = 208)  **Records removed before screening**  Duplicate remove (n=58)  Records marked as illegible by automation tools (n=67)  Records removed for other reasons (n=45)  Records identified from  Organizations (n=3)  Records screened (n =214)  Records excluded (n=56)  Reports sought for retrieved (n=133)  Reports not retrieved (n=32)  Reports assessed for eligibility (n=126)  Reports excluded  Reason1 -Abstract only (n=63)  Reason2 –languege (n=37)  Reason3 -content difference (n=9)  New studies included in the review (n=17)  Reports of new included studies (n=3)  Total studies included in the review (n=20)  Reports of total included studies (n=7)  Reports sought for retrieved (n=3)  Reports not retrieved (n=0)  Reports assessed for eligibility (n=3)  Studies included in the Previous version of reviews (n=7)  **Figure 1.** Flow chart for selection of studies using 2020 PRISMA flow diagram**.**  **4.1. Data Quality Appraisal and Synthesis**  Before inclusion each study in the review of the literature, the quality of each study was assessed by the four authors independently using the Critical Appraisal Skills Programme (CASP) checklists (15, 16),and all authors consulted their findings with each other, then agreed on the final studies to be in the review. The authors defined moderate and high methodological quality as meeting 60-80% and 90-100% of the CASP checklist criteria respectively (15-17). The minimum percentage threshold for inclusion in the review of the literature was decided to be 60% of the criteria (17) and uses the WHO 2011 level of evidence and degree of recommendation (**Table 2**) (18), and this study is registered with a link of <https://www.researchregistry.com/browse-the-registry#registryofsystematicreviewsmeta-analyses/> with a unique identifying number (UIN) of [1318](Manuscript%20on%20%20PE.docx) and the study has high quality based on AMSTAR 2 quality assessment checklist [/https://amstar.ca/Amstar_Checklist.php.](Manuscript%20%20ms.docx)  **Table 2.** WHO 2011 level of evidence and degree of recommendation   \| Level \| Type of evidence \| Degree of recommendation \| \| --- \| --- \| --- \| \| 1a \| Meta-analyses, systematic reviews of RCTs \| Strongly recommended/directly applicable \| \| 1b \| Systematic review \| Highly recommended/directly applicable \| \| 1c \| Randomized clinical trials/RCTs \| Recommended/ applicable \| \| 2a \| Systematic reviews of case-control or cohort studies. \| Extrapolated evidence from other studies \| \| 3a \| Non-analytic studies, e.g. case reports, case series \| Extrapolated evidence from other studies \| | | | 2 |
| Results | 5 | | | A summary of the included studies in the review of the literature can be seen in (Table **1)**. Totally 27 articles were included [guidelines (n=3), Cochrane (=5), systemic reviews (n=7), meta-analyses (=2), RCT (n=4), cohort studies (n=3), and cross-sectional study (n=3) which were more updated and focused on pulmonary embolism management. Illegible articles identified from searches of the electronic databases were imported into the ENDNOTE software version X7.1 (Tomson Reuters, USA) and duplicates were removed. Before findings had begun, full-length articles of the selected studies were read to confirm for fulfilling the inclusion criteria. | | | 1 |
| Discussion | 6 | | | Pulmonary embolism (PE) is a life-threatening condition in which a clot travels from deep veins of the lower extremity to the circulation and lodges into the lungs(7). Clinical presentation of venous thromboembolism (VTE) is globally the third most frequent acute cardiovascular syndrome behind myocardial infarction and stroke (7).  Human immunodeficiency virus (HIV) increases the risk of PE two- to ten fold as compared with the general population, major surgery, Hip or knee replacement, and General anesthesia when compared with epidural (4, 19).  Computed Tomography Pulmonary Angiography (CTPA) has greatly improved the diagnostic approach to patients with suspected PE and is considered to be the reference imaging test, but should be used, with caution in some patients, such as patients with severe renal insufficiency, those with known allergy to contrast media, and pregnant women(19-21). Additionally, ECG findings include sinus tachycardia, atrial dysrhythmia, dramatic shift in R wave axis, incomplete or complete right bundle-branch block, inferolateral ST-segment elevation, or depression, inversion of T waves in leads V1–V4, and biomarkers such as elevated D-dimers or fibrin degradation are suggestive of PE (9, 13, 19, 22, 23). D-dimer assays can rule out PE. But has low specificity of positive tests, especially in older age groups(13, 24).  If the patient presents with at least three parameters out of the five most common signs and symptoms of PE (cough, hypoxia, dyspnea, tachycardia, and chest pain) with the inclusion of X-ray and echocardiography results it is satis­factory pieces of evidence to make high suspicions of acute pul­monary embolism that requires diagnosis and management at bedside within a few minutes, but if the patient is hemodynamically stable, CTPA can be performed to confirm the diagnosis(12, 25).  Acute pulmonary embolism requires anticoagulation to prevent early death and recurrent symptomatic fatal venous thromboembolism. The standard duration of anticoagulation cover at least 3 months and parenteral anticoagulation [unfractionated heparin, low molecular weight heparin, or fondaparinux] over the first 5–10 days should be given to treat acute PE (7, 19, 24, 26, 27).  Hypoxaemia is one feature of severe PE and resulted from the mismatch between ventilation and perfusion, so supplemental oxygen is required in patients' level of SpaO2 <90%. Patients with right ventricular failure are highly susceptible to the development of severe hypotension during induction of anesthesia, intubation, and positive-pressure ventilation (7). Thrombolytic therapy is associated with a significant reduction in overall mortality, pulmonary embolism recurrence as compared with heparin, but increased intracranial hemorrhage and is not significant in hemodynamically stable patients (8, 10, 19, 28, 29).  Hemodynamically deteriorating suspected pulmonary embolism patients require rescue thrombolytic therapy, in addition, surgical embolectomy and catheter-directed treatment are alternatives to the treatment of pulmonary embolism to rescue thrombolytic therapy (7, 9, 19, 24). Intravenous catheter filters reduce the risk of subsequent pulmonary embolism, increase the risk of DVT, and have no significant effect on overall mortality, so Intravenous catheter (IVC) filters should be considered for limited scenarios, such as contraindication to antithrombotic therapy or recurrent pulmonary embolism despite adequate anticoagulation(7, 30). A fixed-dose regimen of rivaroxaban is as effective as standard anticoagulant therapy for the treatment of DVT prophylaxis, without the need for laboratory monitoring(28, 31).  Deep venous thrombosis (DVT) can be prevented through non-pharmacologic prophylaxis ( compression stockings, leg elevation, sequential compression devices (SCDs), ambulation, and vena cava filter) and pharmacologic intervention, which is through the use of blood-thinning medications(2, 11, 27). The most common blood thinner prophylaxis in Ethiopia is unfractionated heparin (UFH) and warfarin. The major side effect of blood-thinning medications is an increased risk of bleeding and some patients are contraindicated for blood-thinning medications since they have a greater risk of developing adverse events(11, 24). The overall mortality rate in untreated patients is 30%, with approximately 10% of patients dying within one hour of the event. Haemodynamically unstable patients have the highest mortality rate, which can be as high as 58%(4, 7, 32). Generally the overall summary for patients with suspected high-risk pulmonary embolism presenting with hemodynamic **stable** and **instable** patients are detailed as shown in (**Figuere2** and **Figure 3**) respectively. | | | 2 |
| Conclusion | 7 | | | All perioperative patients, especially trauma victims, prostate or orthopedic surgery, malignancy, immobility, and obesity; smokers; and oral contraceptive users, antipsychotic medications are at increased risk of venous thromboembolism and need special caution during surgery and anesthesia**.** | | | 2 |
| Funding |  | | | None | | |  |
| Competing interests |  | | | The authors declare that there is no conflict of interest | | |  |
| Availability of data |  | | | Data is available and can be presented upon request time | | |  |

M.J. Page, J.E. McKenzie, P.M. Bossuyt, I. Boutron, T.C. Hoffmann, C.D. Mulrow, et al. The PRISMA 2020 statement: an updated guideline for reporting systematic reviews. International Journal of Surgery (2021):88;105906.

## Annex I: Level of evidence and degree of recommendation

##

| Author | *Year* | *Study design* | \|  \| *No of patients/*  *Studies* \| \| --- \| --- \| | Study intervention | \| *Outcome* \| \| --- \| | | \| *Recommendation* \| \| --- \| |
| --- | --- | --- | --- | --- | --- | --- | --- | --- | --- | --- | --- |
| Konstantinides S V., Torbicki A, Agnelli G, et al | 2014 | Guideline |  | diagnosis and  management of acute pulmonary embolism | | CTPA is the gold standard | \| Strongly recommended \| \| --- \| |
| Hao, Q Br, Dong Yue, J Wu, T Gj, Liu | 2018 | Systematic Review of RCTs | 2197 participants | Effects of thrombolytic therapy for acute PE. | | thrombolytics were better at improving blood flow through the lungs. | Strongly recommended |
| S.Konstantinides, G. Meyer, H.Bueno. et al | 2019 | Guide line |  | diagnosis and management of acute PE | | In suspected PE, perform bedside echocardiography or emergency CTPA | \| Strongly recommended \| \| --- \| |
| Bajc M, Schümichen C, Grüning T , Lindqvist A, Le Roux PY, Alatri A, et al. | 2019 | Guideline |  | Diagnosis of pulmonary embolism. | | The likelihood of PE is assessed using a clinical prediction tool. | Strongly recommended |
| Leibfried M, Falbaum S, Palummo J. | 2018 | Systematic Review | 7897 participants | Treatment of PE | | No difference between oral DTIs and standard anticoagulation | Strongly recommended |
| Raynal P, Cachanado M, Truchot J, Damas-perrichet C, Feral-pierssens A, Goulet H, et al | 2019 | Cohort | 456 patients | Prevalence of PE among ED pts | | prevalence of 2.2%, 95% CI: 1.1–4.3% | Extrapolated evidence from other studies |
| Desciak MC, Martin DE. | 2010 | Review article |  | Perioperative pulmonary embolism | | Surgery puts patients at increased risk for pulmonary  Embolism | Highly recommended |
| Ayalew MB, Horsa BA | 2018 | Crossectional | 206 study subjects | Appropriateness of Pharmacologic Prophylaxis  against DVT | | underutilization of pharmacologic thromboprophylaxis  in medical ward | Extrapolated evidence from other studies |
| Marti C, John G, Konstantinides S, Combescur C, Sanchez O, Lankeit M, et al. | 2015 | Systematic review and meta-analysis |  | Systemic thrombolytic therapy for acute PE | | Thrombolytic therapy induces faster clot dissolution than anticoagulation | Strongly recommended |
| Di Minno, M. N.D.  Ambrosino, P.  Ambrosini, F.  Tremolo, E.  Di Minno, G.  Denali, F. | 2016 | Systematic review and meta-analysis | 22 studies | Prevalence of DVT and PE | | PE is  present in ~ 7% of patients at the time of SVT diagnosis. | Strongly recommended |
| Somasundaram K, Ball J. | 2013 | Review Article |  | Pulmonary embolism and acute severe asthma | | The gold standard for diagnosing PE is multidetector  CTPA | Highly recommended |
| Crawford F, Andras A, Welch K, Sheares K, Keeling D, Chappell FM | 2016 | a systematic review of x sectional | 13 databases | D-dimer test for excluding the diagnosis of PE | | D-dimer test is useful as a rule-out test | Extrapolated evidence from other studies |
| Shonyela FS, Yang S, Liu B, Jiao J. | 2015 | Review article |  | Postoperative acute PE following pulmonary resections | | thrombolytic therapy has been proved to be the best option in treating acute PE | Highly recommended |
| Bikdeli B, Chatterjee S, Desai NR, Kirtane AJ, Desai MM, Bracken MB, et al. | 2017 | Systematic review & meta-analysis | 11 studies | Inferior Vena Cava Filters to Prevent Pulmonary Embolism | | filters appear to reduce the risk of subsequent PE. | Strongly recommended |
| Bailey AL. | 2012 | Article review | 4832 patients | Oral Rivaroxaban for the Trt  Of PE | | a fixed-dose regimen of rivaroxaban alone was not inferior to standard therapy | Highly recommended |
